# The determinants of change in tibial plateau bone area in osteoarthritic knees: a cohort study

**DOI:** 10.1186/ar1726

**Published:** 2005-03-31

**Authors:** Yuanyuan Wang, Anita E Wluka, Flavia M Cicuttini

**Affiliations:** 1 Department of Epidemiology and PreventiveMedicine, Monash UniversityMedical School, Alfred Hospital, Prahran, Vic 3181, Australia; 2 Graduate School of IntegrativeMedicine, Swinburne University of Technology, Hawthorn, Vic 3122, Australia

## Abstract

Bone is integral to the pathogenesis of osteoarthritis (OA). Whether the bone area of the tibial plateau changes over time in subjects with knee OA is unknown. We performed a cohort study to describe this and identify factors that might influence the change. One hundred and twenty-six subjects with knee OA underwent baseline knee radiography and magnetic resonance imaging on their symptomatic knee. They were followed up with a repeatmagnetic resonance image of the same knee approximately 2 years later. The bone area of the tibial plateau was measured at baseline and follow-up. Risk factors assessed at baseline were tested for their association with change in tibial plateau bone area over time. One hundred and seventeen subjects completed the study. The medial and lateral tibial plateau bone areas increased by 2.2 ± 6.9% and 1.5 ± 4.3% per year, respectively. Being male (*P *= 0.001), having a higher body mass index (*P *= 0.002), and having a higher baseline grade of medial joint-space narrowing (*P *= 0.01) were all independently and positively associated with an increased rate of enlargement of bone area of the medial tibial plateau. A larger baseline bone area of the medial tibial plateau was inversely associated with the rate of increase of that area (*P *< 0.001). No factor examined affected the rate of increase of the bone area of the lateral tibial plateau. In subjects with established knee OA, tibial plateau bone area increases over time. The role of subchondral bone change in the pathogenesis of knee OA will need to be determined but may be one explanation for the mechanism of action of risk factors such as body mass index on knee OA.

## Introduction

Osteoarthritis (OA) is the most common form of joint disease, with a prevalence of 10 to 30% in persons over the age of 65 [1]. However, the pathogenesis of this degenerative joint disease is not fully understood. It affects articular cartilage, subchondral bone, and soft tissues including synovium and ligaments. However, the instigating lesion remains unclear and controversial, with proponents for both cartilage and bone abnormalities being the initiating factor [2].

Changes in subchondral bone are well described in established OA, in human [3-7] as well as animal [8-13] models. These changes include remodelling of the subchondral trabeculae [3,4,8-11], stiffening of the subchondral bone [5,12,13], thickening of the subchondral plate [4,6,11], a steep stiffness gradient [5], and a decrease of the ability to absorb energy [7]. These changes affect the mechanical properties of the subchondral bone and have been proposed to play a role in the initiation and progression of degeneration of the overlying articular cartilage [5,13,14]. It has been suggested that the changes in subchondral bone may be the initiating factor in the pathogenesis of OA rather than sequelae of cartilage damage [15]. Changes in the area of subchondral bone may reflect the changes in its architecture. The enlargement of the bone area of the tibial plateau may attenuate the tibial cartilage, and this attenuation may play a role in the process of OA. However, whether the size of subchondral bone is static or changes over time in subjects with OA is unknown.

We studied a cohort of symptomatic subjects with predominantly mild to moderate knee OA over the course of 2 years, to determine whether the bone area of the tibial plateau changes over time and to identify the factors that might influence this change.

## Materials and methods

Patients were recruited by using a combined strategy including advertising through local newspapers and the Victorian branch of the Arthritis Foundation of Australia, as well as collaboration with general practitioners, rheumatologists, and orthopaedic surgeons, as previously described [16]. The study was approved by the ethics committee of the Alfred and Caulfield hospitals inMelbourne, Australia. All the patients gave their informed consent.

One hundred and thirty-two subjects aged over 40 years who fulfilled American College of Rheumatology (ACR) clinical and radiographic criteria for knee OA [17] entered the study. Subjects were excluded if any other form of arthritis was present, if there was any contraindication tomagnetic resonance imaging (MRI), if a total knee replacement was planned, or if they were unable to cooperate with study requirements. We have previously described this population in a study of the determinants of change in the volume of tibial cartilage in knee OA [16]. Weight was measured to the nearest 0.1 kg (shoes, socks, and bulky clothing removed) using a single pair of electronic scales. Height was measured to the nearest 0.1 cm (shoes and socks removed) using a stadiometer. Body mass index (BMI) (weight/height^2 ^in kg/m^2^) was calculated. General health, pain, stiffness, and function were assessed using the SF-36 (36-Item Short-Form Health Survey) [18] and WOMAC (Western Ontario andMcMaster Universities Osteoarthritis Index) [19]. Participants were asked to complete a questionnaire regarding demographic data and current physical activity [20].

At baseline, each subject had a weight-bearing anteroposterior tibiofemoral radiograph taken of the symptomatic knee in full extension. All radiographs were independently scored by two trained observers using a published atlas to classify disease in the tibiofemoral joint [21]. The radiographic features of tibiofemoral OA in each compartment were graded on a 4-point scale (0–3) for individual features of osteophytes and joint-space narrowing [21]. Intraobserver reproducibility was 0.93 for osteophytes and 0.93 for joint-space narrowing. Interobserver reproducibility was 0.86 for osteophytes and 0.85 for joint-space narrowing (by κ statistic) [22]. Where both knees were symptomatic and showed changes of radiographic OA, the knee with the least severe disease was used.

**Figure 1:**
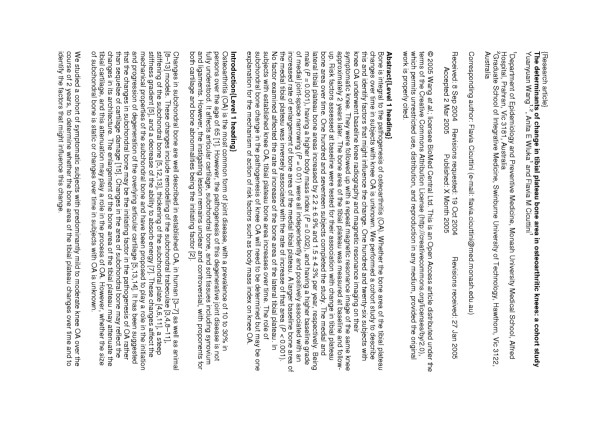
Axial T_1_-weighted fat-saturated 3DMRI image showing measurement of tibial plateau bone area. The area of medial (Roi 2) and lateral (Roi 1) tibial plateau bone is measured manually on the first image that shows both tibial cartilage and subchondral bone (left), and on the next distal image (right). An average of the two areas is used as an estimate of the tibial plateau bone area. MRI, magnetic resonance image; Roi, region of interest.

Each subject had anMRI performed on the symptomatic knee at baseline and approximately 2 years later. Knees were imaged in a sagittal plane on the same 1.5-T whole-body magnetic resonance unit (Signa Advantage HiSpeed GEMedical Systems, Milwaukee, WI, USA) using a commercial receive-only extremity coil. The following sequence and parameters were used: a T_1_-weighted, fat-suppressed 3D gradient recall acquisition in the steady state; flip angle 55 degrees; repetition time 58 ms; echo time 12 ms; field of view 16 cm; 60 partitions; 512 (frequency direction, superior–inferior) × 512 (phase encoding direction, anterior–posterior) matrix; one acquisition, time 11 min 56s. Sagittal images were obtained at a partition thickness of 1.5 mm and an in-plane resolution of 0.31 mm × 0.31 mm (512 × 512 pixels). One trained reader made the measurements in duplicate. The bone areas of the medial and lateral tibial plateaux were determined by means of image processing on an independent work station using the software program Osiris (Digital Imaging Unit, University Hospital of Geneva, Geneva, Switzerland), by creating an isotropic volume from the input images, which were reformatted in the axial plane, and then areas were directly measured from these axial images, as previously described [22-24]. To measure the bone area of the tibial plateau, we selected the first image that showed both tibial cartilage and subchondral bone. The area of medial and lateral tibial plateau bone was measured manually on this image and the next distal image (Fig. 1). An average of the two areas was used as an estimate of the tibial plateau bone area. The coefficients of variation (for the repeated image analysis) for the medial and lateral tibial plateau bone area were 2.3% and 2.4%, respectively [22].

Descriptive statistics for characteristics of the subjects were tabulated. Independent *t*-tests were used for comparison of means. The chi-square test was used to compare nominal characteristics between the groups. Principal outcome measures in analyses were annual change and annual percentage change of tibial plateau bone area. Change in this area was obtained by subtracting the bone area at baseline from that at follow-up. The annual change was calculated by dividing this figure by the time betweenMRI scans. The annual percentage change was obtained by dividing annual change by the baseline bone area and multiplying by 100 to obtain a percentage. Stepwise multiple linear regression techniques were used to explore the possible factors affecting annual percentage change in tibial plateau bone area, including age, gender, BMI, WOMAC score, SF-36 score, physical activity, radiographic features (grades of osteophytes and joint-space narrowing in the studied compartment) and baseline tibial plateau bone area. A *P *value less than 0.05 (two-tailed) was regarded as statistically significant. All analyses were performed using the SPSS statistical package (standard version 11.5.0, SPSS, Chicago, IL, USA).

## Results

One hundred and thirty-two subjects fulfilled the study criteria and entered this study. MR images were available for measurement of tibial plateau bone area in 126 subjects (Table 1); 6MRIs were unavailable for technical reasons. Most subjects had mild to moderate tibiofemoral OA. Compared with women, men were significantly taller (*P *< 0.001) and heavier (*P *= 0.01) and had lower BMI (*P *= 0.02), higher level of physical activity (*P *= 0.01), less severe medial compartment OA (grade 0 to 1 joint-space narrowing in medial compartment) (*P *= 0.04), and larger medial (*P *< 0.001) and lateral (*P *< 0.001) tibial plateau bone area (Table 1). One hundred and seventeen subjects completed the longitudinalMRI component of the study. There were no significant differences between subjects who completed the study and those who did not (results not shown).

The medial tibial bone area increased from 2054.6 ± 363.9 mm^2 ^to 2128.3 ± 370.0 mm^2 ^(*P *< 0.001) and the lateral tibial bone area, from 1407.2 ± 256.7 mm^2 ^to 1442.8 ± 272.4 mm^2 ^over the study period (*P *< 0.001). Medial and lateral tibial plateau bone areas increased by 36.8 mm^2 ^(*P *< 0.001) and 19.1 mm^2^(*P *= 0.004) per year, respectively, representing an annual increase rate of 2.2% and 1.5% (Table 2). Although there were no significant differences between men and women in annual increase or percentage increase of medial or lateral tibial plateau area in univariate analyses, after adjustment for potential confounders (age, BMI, physical activity, grade of osteophytes, grade of joint-space narrowing, and baseline tibial plateau bone area), men showed a significantly greater annual increase of medial tibial plateau bone area than women (*P *= 0.002) and a significantly greater annual percentage increase of medial tibial plateau bone area (5.4% in men compared with 0.004% in women, *P *= 0.001) (Table 2). There were no significant differences between men and women in the change in lateral tibial plateau bone area.

In univariate analyses, a positive association was found between BMI and annual percentage increase in medial tibial plateau area (*P *= 0.002) (Table 3). There was an inverse association between physical activity (*P *= 0.02) and baseline medial tibial plateau area (*P *< 0.001) and annual percentage increase in medial tibial plateau area (Table 3).

In multivariate analyses, gender explained 7.6% of the variance in the annual percentage change in medial tibial bone area; being male was significantly associated with a higher annual percentage increase of medial tibial plateau area (*P *= 0.001), after adjustment for age, BMI, physical activity, grade of osteophytes, grade of joint-space narrowing, and baseline tibial plateau bone area (Table 3). In these analyses, BMI explained 7.3% of the variance in annual percentage change in medial tibial bone area, and grade of medial joint-space narrowing explained 4.6% of variance in annual percentage change in medial tibial bone area. BMI and grade of medial joint-space narrowing were independently and positively associated with the annual percentage increase of medial tibial plateau area (*P *= 0.002 and *P *= 0.01, respectively). Baseline medial tibial plateau area remained inversely associated with the rate of medial tibial plateau area increase (*P *< 0.001) with baseline medial tibial plateau area explaining 19.3% of the variance in annual percentage change in medial tibial bone area. However, the association with physical activity was no longer statistically significant (*P *= 0.40). No significant associations were found between the assessed factors and annual percentage increase of lateral tibial plateau area in multivariate analyses (Table 3).

Adjustment for WOMAC score and SF-36 score did not affect these results (results not shown).

## Discussion

In this 2-year longitudinal study, we found that there was a significant increase in tibial plateau bone area in symptomatic subjects with predominantly mild to moderate knee OA. The rate of increase in medial tibial plateau bone area was greater in men than in women. Higher BMI and higher baseline grade of medial joint-space narrowing were positively associated with an increased rate of enlargement of medial tibial plateau bone area, while a larger baseline medial tibial plateau bone area was inversely associated with the rate of increase of that area. None of the assessed factors were associated with the rate of increase of lateral tibial plateau bone area.

No previous longitudinal studies have examined the change in tibial plateau bone area in subjects with knee OA. In this study, we showed that subjects with symptomatic, mild to moderate knee OA had a significant increase in medial tibial plateau bone area over the 2 years of follow-up. In a cross-sectional study, Jones and colleagues [25] showed that grade 1 medial osteophytosis was associated with a 10 to 16% increase in both medial and lateral tibial bone areas after adjustment for age, sex, and BMI. Similar results have been shown in a study of hip OA, where men with hip OA had larger femoral neck size as assessed byMRI than healthy controls matched for age and sex [26]. In that study, femoral neck size was greater in the hip with higher OA grade [26]. In this study, we found that increasing grade of medial joint-space narrowing was associated with an increased rate of medial tibial plateau bone area increase. However, baseline tibial bone area was inversely associated with the rate of tibial bone area increase. This suggests that the rate of increase of tibial plateau bone area may be more rapid early in knee OA, when the tibial plateau bone area is smaller, and that as the tibial plateau bone area enlarges, the rate of increase slows down. All these associations were demonstrated in the medial compartment of tibial plateau, which is a more common site for knee OA than the lateral compartment [27]. The causes for the differences between the compartments are unknown.

In general, the factors affecting the change in tibial plateau bone area over the period of our observations were consistent with those factors previously described to be associated with tibial plateau bone size in cross-sectional studies [23,25,28,29]. However, in most studies tibial bone size has been measured using different tools [23,25,28-30]. A number of cross-sectional studies have shown that men have larger tibial bone size than women as measured by tibial plateau area [23], bone area at 4% of the tibial length [28], or articular surface area [29]. Although in our longitudinal study we found that BMI was associated with an increased rate of increase in tibial plateau bone area, Dacre and colleagues [30] did not show that BMI was significantly correlated with tibial plateau width measured on radiographs. These differences may be attributable to the different measures used for assessing tibial plateau bone size.

Our measurement of tibial plateau bone area byMRI is averaged on a two-dimensional projection of the tibia. This potential source of systematic error may become especially important when individuals of different body size are compared. The measurement has high reproducibility [22-25]. However, small positional changes in the longitudinal study may have resulted in overestimation of the measurement error and underestimation of longitudinal change. Our results cannot be simply explained by positing an increase in tibial bone size due to the presence of osteophytes, because adjustment for grade of osteophytes did not alter the findings. Indeed, the rate of tibial bone expansion was associated with an increased grade of joint-space narrowing, not of osteophytes. In this study, we measured only bone size and not bone mineral density, which may be important in the initiation and progression of OA [31-33]. It has been well known that normal bone metabolism depends on the presence of vitamin D. Low intake and low serum levels of vitamin D have been shown to be associated with an increased risk for progression of knee OA [34], or increased incidence of radiographically identified hip OA characterized by joint-space narrowing [35]. However, we did not take serum levels or intake of vitamin D into account in this study.

Here we have shown an expansion in medial tibial plateau bone over 2 years in subjects with mild to moderate knee OA. Consistent with this finding, previous human studies have shown changes in density and architecture of the subchondral bone in established OA [3-7]. Animal models support these findings [8-13]. In a guinea pig model of OA, trabecular remodelling was detected deep within the femoral head when only mild cartilage abnormalities were present [8,9]. In the cruciate-deficient-dog model of OA, by the time the dogs were 3 months old the articular cartilage in the unstable knee showed both the histologic changes typical of early OA and loss of trabecular bone [11]. These changes progressed over a period of 18 to 54 months, with subchondral sclerosis and increased thickness of the subchondral plate. By then, the differences in trabecular thickness and in surface-to-volume ratio were greater than at 3 months [11]. These changes affect the mechanical properties of the subchondral bone and may affect the initiation and progression of cartilage degeneration [5,13,14]. However, at this stage it is unclear which abnormality occurs first. In addition, factors, such as knee adduction moment, which have been shown to affect the progression of knee OA, are also associated with increased medial tibial plateau area [36]. This raises the possibility that changes in tibial bone size may mediate the effect of biomechanical factors on the pathogenesis of knee OA. Similarly, it may be that the effect of obesity on risk of knee OA is mediated via an effect on tibial bone.

We have previously reported that bone size was an independent predictor of knee cartilage volume [23]. However, average tibial plateau bone area was not associated with the rate of tibial cartilage loss [16]. Subjects with knee OA experience a loss of tibial cartilage volume, independent of tibial bone size, and an increase of tibial plateau bone area in the knee. This would tend to result in attenuation of articular cartilage over time. This may result in biomechanical changes at the knee as OA progresses, which may further contribute to the pathogenetic process. The mechanism for the bone changes is not known but may be a combination of biomechanical and systemic factors. For example, higher levels of transforming growth factor β and of insulin-like growth factors 1 and 2 may provide a 'bone-forming' stimulus in subjects with OA [37-39], which could explain the larger bone size in OA.

## Conclusion

In subjects with established knee OA, tibial plateau bone area increases over time. The role of subchondral bone change in the pathogenesis of knee OA will need to be determined but may be one explanation for the mechanism of action of risk factors such as BMI on knee OA.

## Abbreviations

BMI = body mass index; MRI = magnetic resonance imaging/image; OA = osteoarthritis; SF-36 = 36-Item Short-Form Health Survey; WOMAC = Western Ontario and McMaster Universities Osteoarthritis Index.

## Competing interests

The author(s) declare that they have no competing interests.

## Authors' contributions

All authors participated in the design of the study. YW carried out the measurement of the tibial plateau bone areas, performed the statistical analysis, and drafted the manuscript. AW and FC reviewed the manuscript. All authors read and approved the final manuscript.

## Figures and Tables

**Table 1:** Characteristics of the study population

	Total (*n *= 126)	Men (*n *= 58)	Women (*n *= 68)	*P*^*a*^
Age, years	63.6 ± 10.1	64.2 ± 10.0	63.2 ± 10.3	0.58
Height, cm	167.7 ± 9.1	174.6 ± 6.8	161.8 ± 6.1	**<0.001**
Weight, kg	81.4 ± 15.5	85.1 ± 14.9	78.3 ± 15.3	**0.01**
BMI, kg/m^2^	28.9 ± 5.1	27.8 ± 3.8	29.9 ± 5.8	**0.02**
WOMAC score	436 ± 224	411 ± 214	457 ± 232	0.25
SF-36 score	98 ± 7	98 ± 6	99 ± 8	0.25
Physical activity	6.2 ± 1.8	6.7 ± 1.7	5.8 ± 1.8	**0.01**
Time between scans, years	1.95 ± 0.21	1.94 ± 0.19	1.96 ± 0.23	0.58
OA grade^b^				
Grade of medial osteophytes				
<2	107	50	57	0.50
≥ 2	18	7	11	0.35
Grade of medial JSN				
<2	90	35	55	**0.04**
≥ 2	35	22	13	0.13
Grade of lateral osteophytes				
<2	103	48	55	0.49
≥ 2	22	9	13	0.39
Grade of lateral JSN				
<2	116	55	61	0.58
≥ 2	9	2	7	0.10
Tibial plateau area, mm^2^				
Medial	2054.6 ± 363.9	2331.1 ± 304.3	1860.2 ± 263.2	**<0.001**
Lateral	1407.2 ± 256.7	1533.4 ± 244.9	1328.2 ± 232.8	**<0.001**

Except where indicated otherwise, values are means ± standard deviations. ^*a*^Men vs women, by independent *t*-tests or chi-square test, as appropriate. ^b^Values are the number of subjects in each grade category. One radiograph was lost after the study commenced. BMI, body mass index; JSN, joint-space narrowing.; MRI, magnetic resonance imaging; OA, osteoarthritis; SF-36, 36-Item Short-Form Health Survey; WOMAC, Western Ontario and McMaster Universities Osteoarthritis Index.

**Table 2:** Annual change and rate in tibial plateau bone area

			Crude analysis			Adjusted analysis		
			
Annual change	Total	*P*	Men	Women	*P*^a^	Men	Women	*P*^b^
Increase, mm^2^								
Medial	36.8 ± 70.7	<0.001	38.3 ± 62.6	35.7 ± 76.1	0.85	70.8 ± 12.3	13.5 ± 9.6	0.002
Lateral	19.1 ± 60.1	0.004	16.5 ± 83.9	20.4 ± 45.1	0.77	17.2 ± 13.3	20.1 ± 8.5	0.87
Change %								
Medial	2.2 ± 6.9	<0.001	1.6 ± 2.8	2.6 ± 8.7	0.49	5.4 ± 1.1	0.004 ± 0.9	0.001
Lateral	1.5 ± 4.3	0.004	1.3 ± 5.8	1.6 ± 3.4	0.79	1.6 ± 0.9	1.5 ± 0.6	0.95

Values are mean ± standard deviations, or means ± standard errors for adjusted analysis. ^a^Men vs women. ^b^Men vs women, adjusted for age, body mass index, physical activity, grade of osteophytes, grade of joint-space narrowing, and baseline tibial plateau bone area.

**Table 3:** Factors affecting annual percentage change in tibial plateau bone area

Tibial plateau area factors^a^	Univariate analysis Regression coefficient (95% CI)	*P*	Multivariate analysis^b ^Regression coefficient (95% CI)	*P*
Medial tibial plateau area				
Age	-0.10 (-0.23, 0.03)	0.13	-0.07 (-0.18, 0.05)	0.26
Gender	0.94 (-1.78, 3.66)	0.49	-5.42 (-8.67, -2.18)	0.001
BMI	0.43 (0.17, 0.70)	0.002	0.42 (0.16, 0.67)	0.002
Physical activity	-0.85 (-1.58, -0.11)	0.02	-0.30 (-1.00, 0.40)	0.40
Grade of medial osteophytes	0.02 (-1.78, 1.83)	0.98	0.27 (-1.53, 2.07)	0.77
Grade of medial JSN	0.35 (-1.10, 1.80)	0.63	1.88 (0.43, 3.33)	0.01
Baseline medial tibial area	-0.006 (-0.010, -0.003)	<0.001	-0.012 (-0.017, -0.008)	<0.001
Lateral tibial plateau area				
Age	-0.02 (-0.10, 0.07)	0.67	-0.01 (-0.11, 0.08)	0.80
Gender	0.25 (-1.62, 2.12)	0.79	-0.08 (-2.51, 2.36)	0.95
BMI	-0.02 (-0.19, 0.15)	0.82	-0.04 (-0.23, 0.16)	0.72
Physical activity	-0.05 (-0.57, 0.48)	0.85	-0.16 (-0.74, 0.41)	0.57
Grade of lateral osteophytes	-0.63 (-1.68, 0.42)	0.24	-0.18 (-1.70, 1.35)	0.82
Grade of lateral JSN	-0.87 (-2.35, 0.61)	0.25	-0.42 (-2.31, 1.48)	0.66
Baseline lateral tibial area	-0.003 (-0.007, 0.001)	0.11	-0.002 (-0.008, 0.003)	0.38

^a^Factors were defined as follows: age = percentage change per 1-year increase in age; gender = men compared with women; BMI = percentage change per unit increase in body mass index; physical activity = percentage change per unit increase in physical activity level; grade of osteophytes = percentage change per increase 1 in grade of osteophytes; grade of JSN=percentage change per increase 1 in grade of joint-space narrowing; baseline tibial area = percentage change per 1 mm^2 ^increase in baseline tibial plateau bone area. ^b^Includes age, gender, BMI, physical activity, grade of osteophytes, grade of JSN, and baseline tibial plateau bone area in regression equation. BMI, body mass index; JSN, joint-space narrowing.
